# Utilization of antenatal care services and its sociodemographic correlates in urban and rural areas in Delhi, India

**DOI:** 10.18332/ejm/140459

**Published:** 2021-09-10

**Authors:** Ruchir Rustagi, Saurav Basu, Suneela Garg, Mongjam M. Singh, Y. M. Mala

**Affiliations:** 1 Department of Community Medicine, Maulana Azad Medical College, New Delhi, India; 2 Department of Obstetrics and Gynaecology, Maulana Azad Medical College, New Delhi, India

**Keywords:** antenatal care, ANC content, adherence

## Abstract

**INTRODUCTION:**

Timely and quality antenatal care (ANC) is an essential element of universal health coverage and a key determinant for the prevention of maternal mortality. Nevertheless, evidence from large-scale health surveys in developing countries highlight a lack of access and utilization of antenatal care especially among socioeconomically disadvantaged populations.

**METHODS:**

A total of 200 women were recruited from urban and rural primary care service provision areas of a government medical college in Delhi during April 2016-2017. Women with infants were interviewed to assess the antenatal care received by them during their recently concluded pregnancy.

**RESULTS:**

The mean (SD) age of the participants was 25.6 (3.9) years. A total of 63 (31.5%) participants were primigravida, and 137 (68.5%) were multigravida. The knowledge of ANC was significantly higher in the participants that were more educated (p<0.001) but it was similar in both the urban and rural sites. Only 107 (53%) participants reported receiving comprehensive antenatal care defined as early registration of pregnancy (within 12 weeks), at least four ANC visits, two doses of tetanus toxoid, and at least 100 days of iron/folic acid supplementation (IFAS). The participants lacking knowledge of ANC had three times higher odds of suboptimal ANC utilization during their previous pregnancy (p=0.018). Furthermore, the ANC content was adequate in terms of medical service provision but deficient in terms of educational and counseling services.

**CONCLUSIONS:**

A high prevalence of suboptimal ANC utilization was observed despite the availability of government-funded primary care.

## INTRODUCTION

Antenatal care (ANC) is the healthcare provided to women who are pregnant, for confirmation and monitoring of the progress of their pregnancy, and to promote their birth preparedness and complication readiness for ensuring optimal birth outcomes for both the mother and her baby^1^. Timely and quality antenatal care is a crucial determinant towards the prevention of maternal mortality, which is a significant developmental goal for developing countries, which contributes to more than 99% of maternal deaths worldwide^2^.

The essential components of quality ANC include early registration of pregnancy, a minimum of four antenatal visits during each pregnancy interspersed over the three trimesters, tetanus toxoid immunization (TTI), and iron/ folic acid supplementation (IFAS)^1^. During antenatal visits, pregnant women should receive appropriate nutrition and health education, undergo clinical and laboratory tests for monitoring maternal and fetal well-being, and evaluated for the early detection of any abnormalities along with their management and referral, as required.

However, the lack of equitable access to quality ANC is a major challenge in resource-limited settings, particularly in developing countries. In India, the second most populous country with the largest reproductive cohort, results of a large-scale nationwide cross-sectional survey (2015-2016) revealed that full antenatal care was provided to only 21% of reproductive age women during their previous pregnancy^3^. Moreover, significant underutilization of ANC has been observed among women living in poverty, low education, poor awareness, cultural traits, and residence in underserved areas^4-6^. Despite considerable advancement in reducing maternal mortality, India is still not on-track to achieve the sustainable developmental goal target of reducing maternal mortality to <100 by 2020 and <70 by 2030^7^.

Identifying the women who are likely to miss receiving complete ANC care during their pregnancy and understanding the causal factors is essential towards the development of effective and targeted public health interventions^8,9^.

The state of Delhi in Northern India has full ANC coverage of only 39% (2015-2016) despite having the highest per capita income in the country and also the highest density of allopathic doctors (161.1 per 100000 population) and nurses in India^3,10^. The present study was, therefore, conducted to estimate comprehensive antenatal care utilization among eligible beneficiaries and their determinants.

## METHODS

### Study design, participants and setting

A cross-sectional study was conducted among postpartum women who had delivered a child within the previous 12 months and were residents of the area for at least 12 months before their delivery. The data were collected for one year from April 2016-2017 at an urban resettlement colony with a population of 22752 and an urbanized village with a population of 5186 in Delhi. These areas comprise the field practice areas of the Department of Community Medicine of a government medical college of the city. The ANC services were provided free of cost at primary care clinics in both the sites through a team of resident doctors from the medical college, and paramedical and auxiliary health staff employed by the government of the National Capital Territory, Delhi. The antenatal women were referred for delivery and specialist care to government-run secondary and tertiary care hospitals within a 5-10 km radius.

### Definitions

Comprehensive antenatal care was defined as early registration of pregnancy (within 12 weeks), at least four ANC visits at a health facility, two doses of tetanus toxoid and at least 100 days of IFAS consumption^1,3,11^. The primary outcome was the proportion of participants who received comprehensive ANC.

### Sample size and sampling

A sample size of 200 was estimated at 95% confidence levels, 6% margin of error, with expected prevalence of comprehensive ANC as 21%^3^, and considering 10% nonresponse. An equal number of participants were included from both the urban and rural area to ensure sample adequacy in terms of representativeness and enable comparisons between both the sites.

In the urban area, the households having mothers of infants were identified with the help of the team of frontline health workers known as Accredited Social Health Activists (ASHAs). Subsequently, a total of 100 households having mothers of infants were selected using the systematic random sampling method. In the rural area, the eligible households were identified through a consecutive house-to-house survey. The study was approved by the Institutional Ethics Committee of the medical college. Written and informed consent was obtained from all the study participants.

The data were collected using a pretested patient interview schedule, including questions adapted from a 2008 UNICEF questionnaire^12^. The face-to-face interviews were conducted in the local language, Hindi, by the first author. The socioeconomic status (SES) of the participants was assessed by calculating their per capita income^13^. Data regarding ANC utilization was validated with the medical records, which were available for 44 women only.

### Statistical analysis

The data were analyzed with IBM SPSS Version 17. Categorical variables were expressed as frequency and proportions and continuous variables as mean and standard deviation for normally distributed data, and median and interquartile range for non-normally distributed data. The differences between the proportions were assessed using the chi-squared test. A p<0.05 was considered statistically significant.

## RESULTS

### Sociodemographic characteristics

A total of 200 women, including 100 each from the urban and rural sites, were recruited in the study. The response rate of the survey was 100%. The mean (SD) age of the participants was 25.6 (3.9) years. A total of 122 (61%) participants were natives, while 78 (39%) were migrants, and all were married. There were 93 (46.5%) living in nuclear families and 107 (53.5%) in intergenerational families. The educational level of the participants was: illiterate 34 (17%), middle school 56 (28%), and high school and above in 110 (55%) participants. Most (94%) participants were not employed and lived as homemakers.

### Obstetric characteristics

A total of 63 (31.5%) participants were primigravida, and 137 (68.5%) were multigravida. The median number of children borne by the participants was 2 (IQR: 1-2). A history of abortion during past pregnancies was reported by 62 (31%) participants, of which 52 (83.9%) were spontaneous, and 10 (16.1%) were induced. The mode of delivery was normal vaginal in 167 (83.5%) and cesarean section in 33 (16.5%) participants. Institutional delivery was undertaken by 188 and home delivery by a trained midwife in 12 cases (7 in urban and 5 in the rural area).

### Knowledge regarding ANC

Knowledge regarding the frequency of antenatal visits was present in 44 (22%) participants, and that regarding the benefits of IFAS was correctly reported by 75 (37.5%) participants. The knowledge was higher in the participants that were more educated (p<0.001), living in a joint family (p = 0.04), aged >25 years (p<0.001) and native residents compared to migrants (p<0.01), but it was similar in both the urban and rural sites.

### Utilization of ANC services

Government healthcare facilities were preferred for obtaining ANC by 151 (75.5%) participants, and private facility in the others (24.5%). A total of 131 (69.3%) participants were registered at an ANC clinic within their first trimester of pregnancy, with no significant rural-urban variation (p = 0.50). There were 30 (15%) participants who received <4 antenatal visits during their period of pregnancy. ANC services provided were nearly complete (97.5%) in terms of tetanus toxoid vaccination and provision of IFAS (100%). However, the consumption of IFA tablets for >100 days during their previous pregnancy was reported by 172 (86%) participants only, among whom 30 (18.4%) participants also reported an irregular pattern of IFAS intake.

A total of 107 (53%) participants reported receiving comprehensive antenatal care. On bivariate analysis, participants with at least 10 years of education for both the woman and her husband, higher socioeconomic status, multigravida, and living in a joint family were more likely to have received comprehensive ANC during their previous pregnancy. However, none of these factors was statistically significant. The participants having knowledge of both, the frequency of ANC visits and benefits of IFAS, had significantly higher odds of having received comprehensive ANC during their previous pregnancy (p = 0.018) (Table 1).

**Table 1:** Sociodemographic characteristics of parents (N=200)

*Characteristics*	*Total n (%)*	*Comprehensive ANC not received (n=93) n (%)*	*OR (95% CI)*	*^p^*
**Site**				
Urban	100 (50)	49 (49.0)		
Rural	100 (50)	44 (44.0)	1	0.571
**Age** (years)				
<25	110 (55.0)	50 (45.4)	1	
>26	90 (45.0)	43 (47.8)	0.91 (0.52-1.59)	0.777
**Education** (years)				
<10	90 (45.0)	47 (52.2)	1.52 (0.87-2.66)	
>10	110 (55.0)	46 (41.8)	1	0.156
**Husband's education** (years)				
<10	64 (32.0)	35 (54.7)	1.62 (0.89-2.95)	
>10	136 (68.0)	58 (42.6)	1	0.129
**SES**				
High (Class I-III)	140 (70.0)	71 (50.7)	0.56 (0.3-1.05)	0.089
Low (Class IV-V)	60 (30.0)	22 (36.7)	1	
**Family type**				
Joint	107 (53.5)	45 (42.0)	1	
Nuclear	93 (46.5)	48 (51.6)	1.4 (0.84-2.57)	0.202
**Gravida**				
Primi	63 (31.5)	24 (38.1)	1	0.127
Multi	137 (68.5)	69 (50.4)	1.65 (0.90-3.0)	
**Number of abortions**				
0	138 (69.0)	63 (37.5)	1	
>1	62 (31.0)	30 (48.4)	1.11 (0.61-2.0)	0.760
Knowledge of ANC*				
Good	25 (12.5)	6 (24.0)	1	0.018
Poor	175 (87.5)	87 (49.7)	3.1 (1.19-8.20)	

* Knowledge of both, the number of ANC visits required, and benefits of IFAS during pregnancy.

### ANC content

Most participants underwent regular medical examination in terms of blood pressure monitoring (93.5%), abdominal examination (94%), weight measurement (93.5%), laboratory investigations (91.5%) and ultrasound (93%). Nevertheless, the services in terms of educational and counseling services including birth preparedness and complication readiness (24%), physical activity (62%), family planning (63%) and dietary advice (72.5%), were suboptimal.

## DISCUSSION

The achievement of universal maternal health coverage is a key public health agenda in the developing world^10^. Comprehensive ANC utilization was lacking in nearly half (47%) of the participants. The deficiencies in ANC content were particularly related to inadequate counseling, complication readiness, dietary advice, and physical activity. The ANC related internal examination was incomplete in a significant proportion of the participants. Nevertheless, the comprehensive ANC coverage observed in the study areas was significantly higher than the extent of full ANC coverage reported in the entire Delhi state (39%)^3^. Data from the fourth round of the National Family Health Survey showed that lower maternal education was associated with lower odds of full ANC utilization among Indian women^14^. However, in this study, lower maternal education correlated with reduced awareness of ANC, which in turn was associated with suboptimal ANC utilization.

The evidence from the study has some important implications for the Indian Reproductive and Maternal Newborn Child Health Programme. First, most women in economically advanced states in India, like Delhi, are likely to receive an adequate number of ANC visits, but this may be inadequate for fulfilling the health needs and empowerment of the vulnerable pregnant women. Consequently, there is an urgent need for oversight and policy interventions for bridging these healthcare gaps^15^. Second, in the present study, traditionally underserved areas, including an urban resettlement colony and a village, had significantly higher ANC coverage compared to the state average. The higher ANC coverage in this study was likely because of the ease of access in obtaining ANC at the primary care facilities functioning in these areas, highlighting the need for policy efforts towards strengthening primary healthcare. Previous studies in similar settings have also reported the correlation of the extent of ANC coverage with the robustness of the primary healthcare availability^16,17^.

### Limitations

There are certain study limitations. First, the study was conducted in two sites, with a small sample size, which may not be generalizable to divergent health settings. Second, there is the chance of recall bias as less than one in four participants could produce their medical records related to ANC during their previous pregnancy, indicating the need for digitization of health records. Finally, we did not assess the appropriateness of the timing of the ANC attendance in the participants beyond the initial registration.

## CONCLUSIONS

Lower maternal education correlated with reduced awareness of ANC, which in turn was associated with suboptimal ANC utilization. A high prevalence of suboptimal ANC utilization was observed despite the availability of government-funded primary care.

## Data Availability

The data supporting this research cannot be made available for privacy reasons.
